# A Case of Encysted Placenta, from the Hour-Glass Contraction of the Uterus, the Placenta Being Unnaturally Attached to the Fundus Uteri by a Dense Cord of Cellular Substance

**Published:** 1845-11

**Authors:** M. H. Fee

**Affiliations:** Leatherwood, Indiana


Art. VI.—*A Case of Encysted Placenta, from the Hour-glass Contraction of the Uterus, the Placenta being unnaturally attached to the Fundus Uteri by a dense cord of cellular substance.* By Dr. M. H. Fee, of Leatherwood, Ia.

I was called to Mrs. H. on the morning of the 21st inst.; she had been delivered of a healthy full-grown male child, about 10 o'clock the previous night. Everything up to the birth of the child had gone on well; immediately after the birth, flooding commenced, and the midwife in attendance found that something unusual was the matter, but she knew not what; she could not deliver the placenta, although the pains were of the best character. The flooding continued violently, and threatened to prostrate the vital powers of the body in a very short time, unless she obtained relief. The midwife became alarmed, and insisted that I should be sent for. In accordance with her desire, I was sent for and arrived at the lady's residence at 7, A. M., the morning after the birth of the child.

I found her almost dead from loss of blood, having, as the females present informed me, lost almost a gallon. I found her pulse 136 and small; surface cold and clammy; respiration 40 in a minute and feeble; excessive soreness of the vagina and uterus, preventing examination per vaginam. I gave her immediately a pill of gum opii 1½ gr., acet. plumb. xi grs., which in an hour controlled the flooding considerably. At the expiration of the hour, I gave another pill of similar composition, which at the end of another hour completely arrested the flooding, and reduced the morbid sensibility of the parts sufficiently to allow of an examination. By it I ascertained that the uterus was so firmly contracted at its middle as scarcely to allow the passage of a finger. I gave her immediately xv grains of ergot, which I repeated in thirty minutes; at the same time I touched the contraction with my finger wet with a solution of hyoscyamus, repeating this often. In less than an hour, I was gratified to find that the ergot was acting promptly upon the longitudinal fibres of the uterus, causing them to contract forcibly; while the action of the hyoscyamus, on the circular fibres was equally favorable. In a few minutes the placenta was forced through the stricture into the vagina. I now thought the difficulty over, and taking hold of the cord made gentle traction, requesting the lady to assist me as much as her strength would permit. To my astonishment I found that at every traction of the cord, there was a corresponding dip of the uterus into the lower pelvis; this led me to suspect unnatural adhesions. To satisfy myself I endeavored to pass my finger beyond the placenta, but I failed in this owing to its prodigious size. I was at a loss what to do; but finally concluded, that as the lady was in an improving condition, and in no particular danger from delay, I would leave the case for a short time in the hands of nature, until I could cast in my mind, what was best to be done. This resolution had scarcely been complied with, when nature commenced her operations in a quarter I had not anticipated. The patient commenced vomiting violently, from indigestible food which she had eaten the day prevous at dinner. During her excessive efforts in vomiting, the placenta was expelled; thus in the simplest manner performing in a short time, what might have required me hours to effect.

Upon examination of the placental mass, I found it at least twice the usual size; at the centre of the uterine surface, I found a very short cord of tolerably dense cellular substance, one and a half inches in diameter, very elastic, and broken obliquely across. The uterus contracted firmly, the lady rallied her powers, and at the present time is doing well. The time which elapsed, from the birth of the child, until the expulsion of the placenta, was 15 hours.

If I should ever meet with a case again, in which I had reasonable grounds to believe there was unnatural adhesion of the placenta, I would without hesitation administer an emetic, in thirty minutes after a full dose of ergot.

August 27th, 1845.

